# Recombinant spider silk from aqueous solutions via a bio-inspired microfluidic chip

**DOI:** 10.1038/srep36473

**Published:** 2016-11-07

**Authors:** Qingfa Peng, Yaopeng Zhang, Li Lu, Huili Shao, Kankan Qin, Xuechao Hu, Xiaoxia Xia

**Affiliations:** 1 State Key Laboratory for Modification of Chemical Fibres and Polymer Materials, College of Materials Science and Engineering, Donghua University, Shanghai, 201620, China; 2 School of Life Sciences and Biotechnology, Shanghai Jiao Tong University, Shanghai 200240, China

## Abstract

Spiders achieve superior silk fibres by controlling the molecular assembly of silk proteins and the hierarchical structure of fibres. However, current wet-spinning process for recombinant spidroins oversimplifies the natural spinning process. Here, water-soluble recombinant spider dragline silk protein (with a low molecular weight of 47 kDa) was adopted to prepare aqueous spinning dope. Artificial spider silks were spun via microfluidic wet-spinning, using a continuous post-spin drawing process (WS-PSD). By mimicking the natural spinning apparatus, shearing and elongational sections were integrated in the microfluidic spinning chip to induce assembly, orientation of spidroins, and fibril structure formation. The additional post-spin drawing process following the wet-spinning section partially mimics the spinning process of natural spider silk and substantially contributes to the compact aggregation of microfibrils. Subsequent post-stretching further improves the hierarchical structure of the fibres, including the crystalline structure, orientation, and fibril melting. The tensile strength and elongation of post-treated fibres reached up to 510 MPa and 15%, respectively.

Spider dragline silk has extraordinary mechanical properties, such as good strength, superior flexibility and exceptional toughness^1^. These properties are attributed to the hierarchical structure of silk fibres as well as the ingenious amino acid sequence of spidroins. Earlier studies revealed natural spider dragline silk to be composed of a large number of small crystallites, separated by amorphous regions made of rubber-like chains^2^. Furthermore, the crystalline region is composed of highly oriented, alanine-rich crystals of β-sheets and weakly oriented, crystalline un-aggregated sheets^3^. Confined nano-crystallites connected via weak hydrogen bonds provide spider dragline silk with exceptional toughness, while intra-molecular unfolding significantly contributes to the strain hardening of dragline silk^4^,^5^. These structural features originate in the alternative sequence of the amorphous, glycine-rich domains and the crystalline, alanine-rich domains of natural spider silk^6^. In addition to the amino acid sequences of the protein, the spinning process is another key factor affecting the structure formation of spider silk. The composition of the ‘raw’ protein spinning solution as well as the spinning process are both considered to be essential for successful spider dragline silk production^7^,^8^,^9^.

Due to spider cannibalism, it is not possible to obtain sufficient amounts of natural spider silk protein. However, regenerated silk fibroin (RSF) can be easily obtained from *Bomby mori* silkworm larvae. Dissolved cocoons were commonly used as raw material for bio-mimicking the spinning process of silkworm or spider^10^,^11^. However, the different sequences of RSF compared to spider silk may restrict the mechanical performance of artificial silk. Thus, recombinant spider dragline silk proteins with similar amino acid sequences as their natural counterparts were produced in heterologous hosts^12^,^13^,^14^,^15^. Moreover, the function of C-terminal and N-terminal domains of the recombinant spider silk proteins have been investigated extensively^16^,^17^. To prepare artificial spider silk, various recombinant spidroins have been substituted as feedstocks in many recent studies. However, the mechanical properties of the resulting fibres, spun from recombinant spidroins with low molecular weight (MW) are disappointing^18^,^19^,^20^. Artificial fibres of large proteins composed of alanine-rich and glycine/proline-rich motifs display adequate toughness, but poor tensile strength^14^,^15^. Moreover, the recombinant spidroins with high MW are not soluble in aqueous solutions at high concentration and most of the protein variants are typically dissolved in hexafluoroisopropanol and coagulated in either methanol or isopropanol. These denaturing solvents may result in an incorrectly folded secondary structure and thus, disappointing mechanical properties^21^.

Close examination of spider and silkworm silk glands reveals that both spinning apparatuses are essentially complex microfluidic systems; therefore, microfluidics have attracted significant attention to spin artificial silk from RSF solutions^22^,^23^. Furthermore, the presence of an elongational flow in microfluidics has been demonstrated to be essential for the assembly of recombinant spider proteins^24^. According to Knight^25^, the diameter of the tapered S-duct of *Nephila edulis* decreases as a two-stage hyperbolic curve from the funnel up to the draw down taper. Here, the diameter decreases more rapidly, following a two-stage exponential function. However, the specific function of the hyperbolic curve and exponential draw down taper were not clear until now. Compared to the geometry of the spider silk gland, however, the geometry of the *Bombyx mori* silkworm silk gland has been investigated more extensively^26^. The silk gland in the silkworm and major ampullate (MA) gland in the spider are both funnels with decreasing diameters. Despite fundamental differences in composition and structure of spider MA and silkworm fibroin proteins, the silk glands of both organisms have been shown to function similarly. In both glands, highly concentrated spinning dope flows through the contracted geometry of the silk gland and is exposed to ionic gradients that regulate its state of crystallization by forming liquid crystalline and inducing protein conformational changes and aggregations^7^,^10^,^27^. Considering the similarity of the contracting geometry and function of the silkworm and spider glands, we designed biomimetic microfluidic spinning channels (Fig. 1a), emulating the specific geometry found in silkworm silk glands by Asakura *et al*.^26^.

In this study, the recombinant MA spidroin I (MaSp1) of the spider *Nephila clavipes* (and with a molecular weight (MW) of 47 kDa) was expressed in metabolically engineered *Escherichia coli* and subsequently used as feedstock for spinning dope. The relatively small MW makes the recombinant spidroin soluble and stable in water, avoiding the use of denaturing organic solvents. Two different spinning processes were applied, using a bio-inspired microfluidic chip. One of these processes is microfluidic wet-spinning (WS), which uses 100% ethanol as coagulation bath (Fig. 1b). The other process is a modified wet-spinning (WS-PSD), in which a continuous post-spin drawing process was added in air after the as-spun fibre was drawn out of the ethanol coagulation bath (see Fig. 1c). The additional step in air, which is also necessary for the spinning process of the spider, induces the re-assembly of protein in air. Surprisingly, if done this way, fibre yielded is much more compact and homogeneous compared to microfluidic wet-spinning.

## Results

### Effects of biomimetic spinning conditions

**Figure 1:**
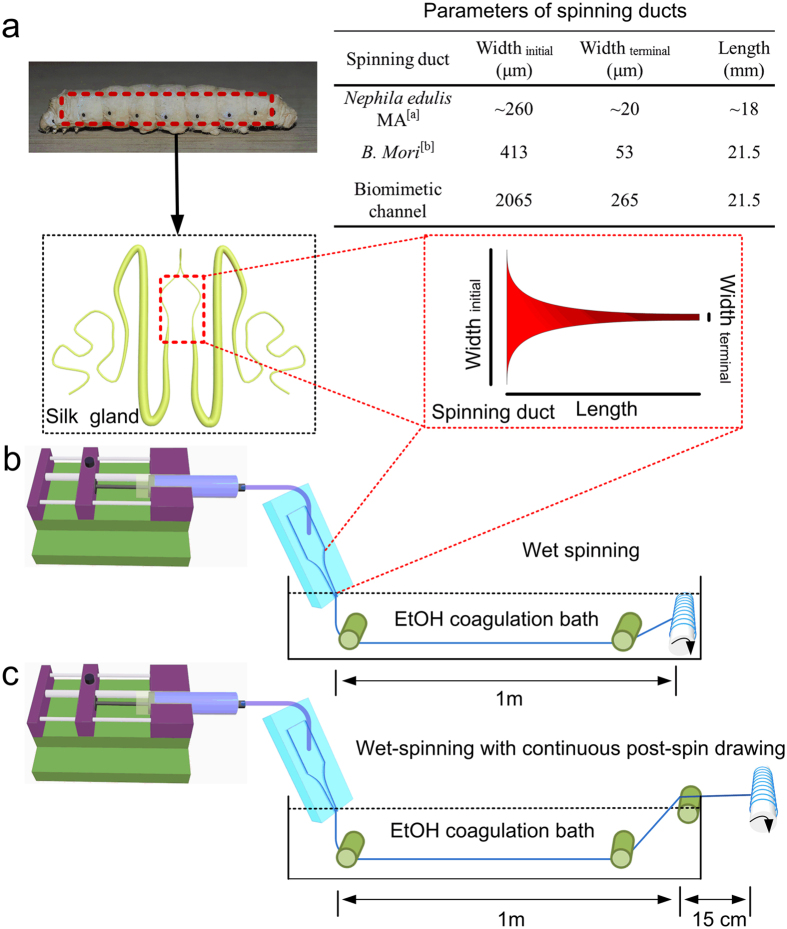
Schematics of the microfluidic spinning process of recombinant spider dragline silk. **(a)** Geometries of the spinning ducts of spider and silkworm. Both contracting spinning ducts enable the proteins to be compact and orderd under shearing and elongation forces. A biomimetic, microfluidic channel was designed to emulate the specific geometry of the silkworm silk gland. **(b)** Wet-spinning process (WS). **(c)** Wet-spinning process with continuous post-spin drawing in the air (WS-PSD). ^a^The diameter of the tapered *N. edule* S-duct decreases as a two-stage hyperbolic curve from the funnel, up to a zone called the draw down taper, where the diameter decreases more rapidly, following a two-stage exponential function^25^. ^b^The diameter (Y) of the lumen of the silkworm (*B. mori*) duct decreases following a second-order exponential regression line with distance from the start of duct (X). Regression line Y = A(1/(1 + exp(BX)) + C(1/(1 + exp(DX)), where A = 238, B = 6.18E-05, C = 588, D = 0.003, and R^2^ = 0.988^26^.

**Figure 2:**
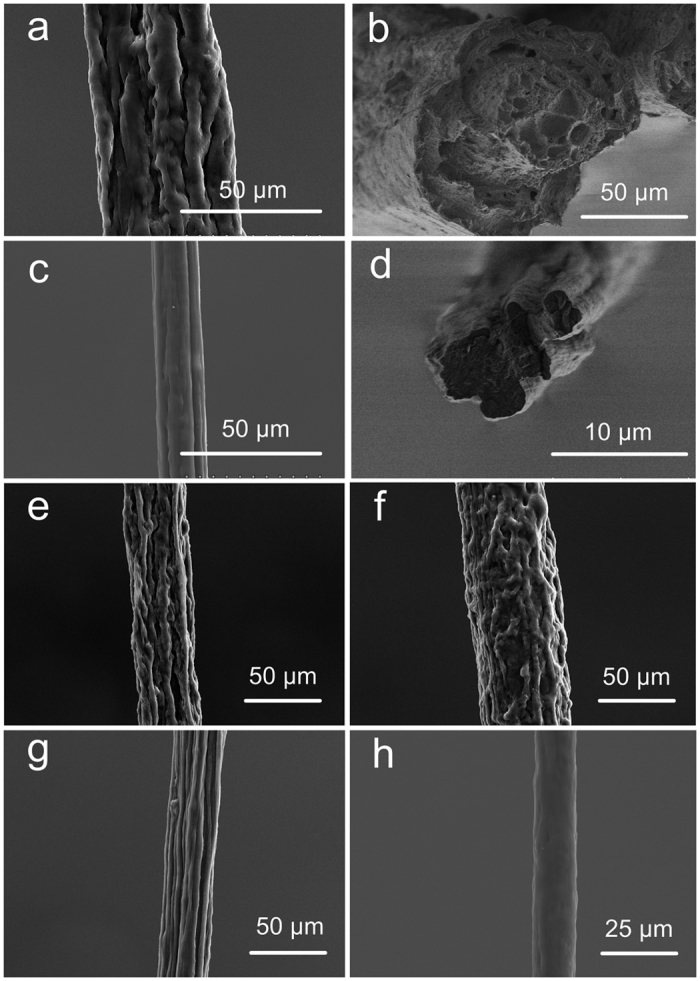
SEM images of recombinant spider silks. (**a**) Surface of as-spun WS, **(b)** cross-section of as-spun WS, **(c)** surface of as-spun WS-PSD, **(d)** cross-section of WS-PSD, and surfaces of **(e)** WS-1x, **(f)** WS-1x-h, **(g)** WS-3x, and **(h)** WS-PSD-3x.

During the wet-spinning process, recombinant spidroin dope was subjected to shearing forces along the microfluidic channel, before it was extruded into the ethanol coagulation (Fig. 1b). SEM shows as-spun WS fibre to be composed of aggregated spidroin fibril bundles along the fibre axis. The fibrillar structure has been reported both in natural spider dragline silk and recombinant dragline silk^10^,^12^. However, as-spun WS fibres show noticeable cracks and granules on the fibre surface (Fig. 2a), as well as many irregular voids and pores in the cross section (Fig. 2b).

Compared to the WS process, the WS-PSD process shown in Fig. 1c uses an additional post-spin drawing process in air. The drawing process in air has normally been applied in the forced reeling of natural spider dragline silk^8^,^9^, natural silkworm silk^28^,^29^, and in the production of RSF silk^30^,^31^. Recently, Copeland *et al*. prepared synthetic spider silk using a wet-spinning process that involved an air gap^32^. However, the function of this air gap was not investigated. After the additional post-spin drawing process in air, the resultant as-spun WS-PSD fibres exhibit dramatically improved morphology compared to the WS counterpart (Fig. 2c,d). A more compact structure, smoother surface, and smaller diameter of the as-spun WS-PSD fibre could be obtained (Fig. 2c). Furthermore, the cross-section of the fibre becomes denser in the absence of voids and pores (Fig. 2d).

### Fibre morphology of post-treated fibres

Subsequent to immersion in a 70 vol. % ethanol aqueous solution for five seconds to partially dissolve NaCl, the water-soluble as-spun WS fibre changed into a water insoluble fibre (WS-1x), showing no evident changes in fibre morphology (Fig. 2e). WS-1x was then immersed in water for a total of 1 min to further dissolve NaCl as much as possible. The WS-1x-h fibre treated with water had a more compact fibril structure (Fig. 2f) and was stable in both water and air. WS-3x and WS-PSD-3x fibres were obtained from the as-spun WS and WS-PSD fibres via post-stretching thrice at 0.9 mm s^−1^ in 70 vol % ethanol aqueous solution, respectively. WS-3x shows a highly oriented fibril structure, however, there were obvious longitudinal cracks on the fibre surface (Fig. 2g). The cracks were likely caused by elongated fibril bundles and dissolution of NaCl in 70 vol % aqueous solution during the stretching process. In contrast, WS-PSD-3x (Fig. 2h) features increased homogeneity and is smoother than WS-PSD (Fig. 2d) as well as all other wet-spun fibres.

### Structure of recombinant fibres

**Figure 3:**
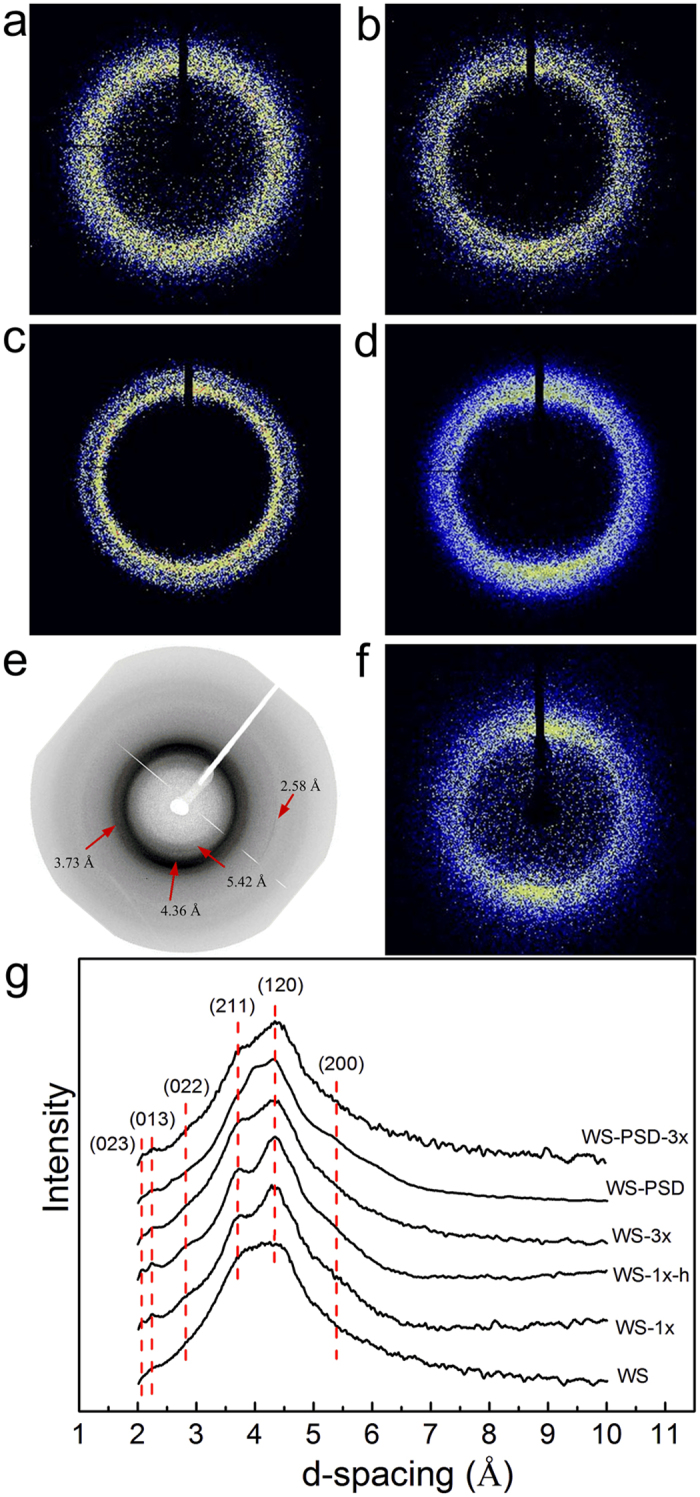
Crystalline structures of recombinant spider silk fibres. **(a)** WS, **(b)** WS-1x, **(c)** WS-1x-h, **(d)** WS-3x, **(e)** WS-PSD, **(f)** WS-PSD-3x, and **(g)** corresponding 1D diffractograms.

Figure 3 shows WAXD patterns and corresponding 1D diffractograms of recombinant spider dragline silk fibres. A two-dimensional WAXD pattern of WS shows a diffusion halo and a 1D diagram has no obvious diffraction peak (Fig. 3a,g). This indicates that WS mainly comprises a disordered and imperfect crystal structure, leading to solubility in water. Most of the WS filament was evidently dissolved in water after 10 s, whereas it cannot be dissolved in 70% ethanol aqueous solution. Subsequent to fibre immersion in ethanol aqueous solution (WS-1x), the diffusion halo was divided into two diffusion rings (Fig. 3b). The corresponding 1D diffractogram (Fig. 3g) shows two obvious diffraction peaks at the position of the (120) reflection (d = 4.36 Å) and the (211) reflection (d = 3.73 Å). Matching results have also been observed for spider dragline silk^33^. This indicates that the post-treatment process results in improved crystal structures similar to natural spider silk. These results show that further water treatment (WS-1x-h) promotes the formation of a finer crystal structure with clear diffraction peaks at (120) and (211) lattice planes (Fig. 3c,g). These results further indicate water molecules to activate the movement of molecular chains via diffusion into the amorphous regions and promoting the intermolecular interactions via dissolving NaCl.

However, 2D WAXD patterns of WS-PSD (as-spun fibre) show distinguishable diffraction arcs at (200) and (120) lattice planes along the equatorial direction, indicating that crystalline region was oriented along the fibre axis during the post-spin drawing process in air (Fig. 3e). The diffractograms of the post-stretched fibres WS-3x and WS-PSD-3x (Fig. 3d,f) reveal that crystalline regions were highly aligned along the fibre direction upon post-stretching.

**Figure 4:**
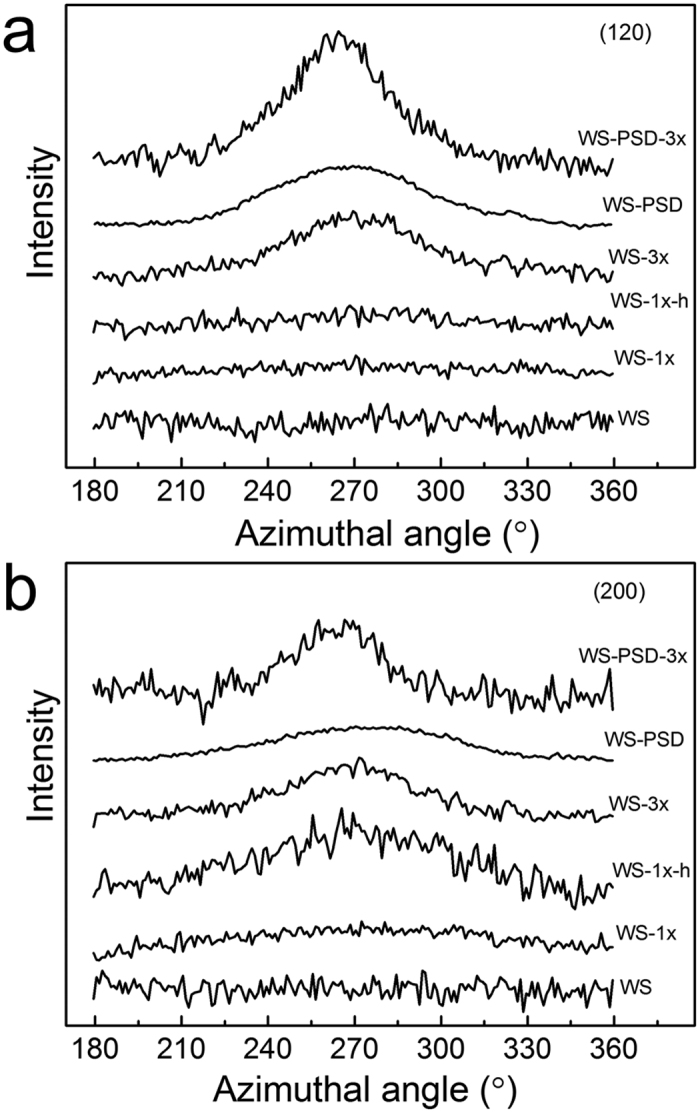
X-ray intensity of recombinant spider silk fibres as a function of azimuth angle at lattice planes of **(a)** (120) and **(b)** (200).

The diffraction patterns reveal a semi-crystalline structure with nanocrystallites embedded within the amorphous matrix similar to natural dragline silk^33^. The crystalline and amorphous orientation along the thread axis can be characterized via orientation factors *f*_c_ and *f*_a_, respectively^33^. The 1D azimuthal intensity profile of the radially integrated (120) and (200) peaks is shown in Fig. 4. Compared to all post-treated WS fibres, WS-PSD-3x evidently show smaller full width at half-maximum of the peak (FWHM), indicating higher crystalline orientation along the fibre axis. Moreover, the as-spun WS-PSD also shows smaller FWHM compared to the as-spun WS.

Table 1 provides details about the crystalline structures of the fibres. The crystallinity, crystalline orientation, and crystal sizes of WS-PSD are higher compared to WS, indicating that post-spin drawing in air promotes both formation and orientation of the crystalline structure. Following further post-stretching treatment, WS-PSD-3x shows higher crystallinity and smaller crystal size. Compared to the disordered crystalline structure of WS, post-stretching drastically increased the crystalline orientation of WS-3x. However, WS and WS-3x do not show noticeable changes in crystallinity and crystal size. This may be ascribed to the incompact and inhomogeneous fibre structure, impeding intermolecular interactions to form crystals with fine structures.

Infrared spectra were analysed to determine the secondary structure composition of the recombinant spider silk fibres. Details of this structure assessment can be found in Supplementary Fig. 2. Table 1 also lists the contents of secondary structures of recombinant dragline silk fibres. Immersion of WS in 70% ethanol water solution clearly increased the β-sheet content of WS-1x. The β-sheet content of WS-PSD is equally high as that of WS-1x, much higher compared to WS. This indicates that post-spin drawing in air induced the β-sheet structure formation by activating the molecular movement of the amorphous regions. The β-sheet content of WS-PSD-3x is noticeably increased by post-stretching treatment. However, the β-sheet contents of WS-1x-h and WS-3x do not increase during water treatment and post-stretching. This indicates that post-stretching may induce the formation of the β-sheet structure via intermolecular hydrogen bonds in the fibril bundles of WS-PSD. However, the exceptionally short post-treatment for WS in 70 vol % ethanol aqueous solution crystallizes WS-1x. Low mobility of “frozen” spidroin molecules impedes further formation of the β-sheet structure.

The birefringence index demonstrates the orientation of molecular chains along the fibre axis. For natural spider silk^34^, an inverse relationship was reported between fibre diameter and the birefringence calculated from optical retardations and diameters. For regenerated silk fibroin fibre, which is created via dissolving silk fibres (commonly silkworm silk cocoons) in chemicals and restructuring the solution into fibre, a similar relationship was found^31^. Moreover, breaking stress of the RSF fibres increased with the increasing birefringence^31^. Upon drawing, the birefringence of silks is positively correlated with molecular orientation, resulting in an increase in β-sheet crystalline area proportion and amorphous domains^20^. The birefringence index of wet-spun fibres does not change noticeably after post-treatment and post-stretching. However, the birefringence index of WS-PSD-3x is about four times higher compared to that of WS-3x (see Supplementary Fig. 3). Given the results of crystallinity and β-sheet content, this indicates that the orientation of the protein molecular chains of WS-PSD-3x is promoting the formation of ordered β-sheet structures.

### Mechanical properties of recombinant fibres

**Figure 5:**
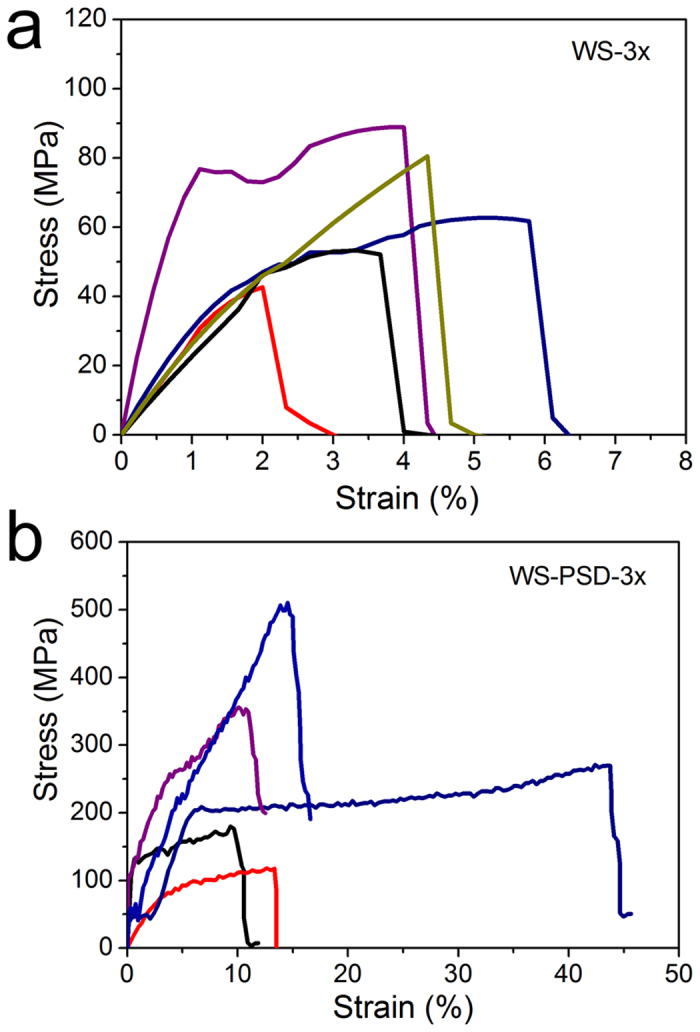
Mechanical properties of recombinant fibres. Strain-stress curves of recombinant spider silk fibres of **(a)** WS-3x and **(b)** WS-PSD-3x.

Figure 5 depicts stress-strain curves of WS-3x and WS-PSD-3x. The mechanical properties of fibres were compared with recombinant spider silk and with literature values of natural spider dragline silk (Table 2). The average breaking strength and strain of WS-3x was 62.3 MPa ± 17.2 MPa and 3.5% ± 1.2%, respectively. Compared to WS-3x, WS-PSD-3x shows a significant increase in extensibility and strength, which is 18.3% ± 12.8% and 286.2 MPa ± 137.7 MPa in average, respectively. Maximal breaking strength was up to 505 MPa and the largest strain was up to 44% (Fig. 5b).

Compared to WS-3x, WS-PSD-3x shows considerably increased modulus and toughness. For native-sized (284.9 kDa) recombinant spider dragline silk with a post-draw ratio of five, breaking strength and breaking elongation of up to 508 MPa and 15% were observed, respectively^12^. Biomimetic spinning of recombinant garden spider protein (120 kD) yielded highly flexible silk fibre with a breaking strength of 383 MPa following post-stretching of up to 600% of the initial length^15^. Large recombinant egg-case silk protein (378 kDa) was spun into homogeneous fibres with breaking strength and strain of 308 MPa and 10%, respectively^14^. Although much smaller spidroins were used compared to the building blocks in this study, WS-PSD-3x shows comparable mechanical properties to the reported recombinant spider dragline silk. However, our spinning dope is an aqueous solution and the spinning process is thus economic and non-toxic.

**Figure 6:**
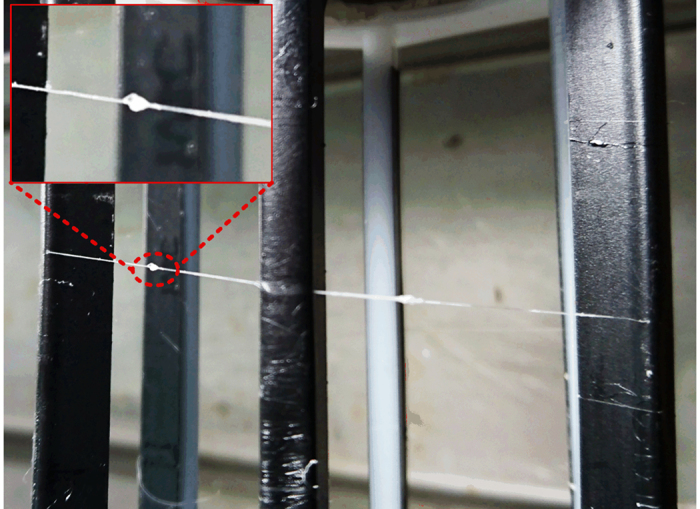
Macroscopic image of recombinant spider silk obtained via microfluidic wet-spinning with continuous post-spin drawing (WS-PSD). Insert shows irregular aggregations, which might be NaCl precipitated from fibre.

Unfortunately, the data of WS-PSD-3x mechanical property exhibit relatively large variability. We suspect this variability to be attributed to the inhomogeneity of NaCl within the fibre, as well as the molecular structure variation of spidroin. As can be seen from a macroscopic image of the WS-PSD spinning process (Fig. 6), some irregular aggregation exists on the surface of the fibre, which was reeled in air subsequent to leaving the ethanol coagulation bath. We suspect that it is not possible to form irregular aggregation via Raleigh instability droplet formation on fibres. First, WS and WS-PSD were coagulated in ethanol subject to equal conditions. Following coagulation, the spinning dope has been solidified and cannot flow to form instable droplets in the subsequent post-drawing process in air. No such aggregation could be found on the surface of WS as spun fibre compared to WS-PSD. However, it may be possible for small NaCl molecules to move once water molecules were adsorbed into the WS-PSD fibre.

**Figure 7:**
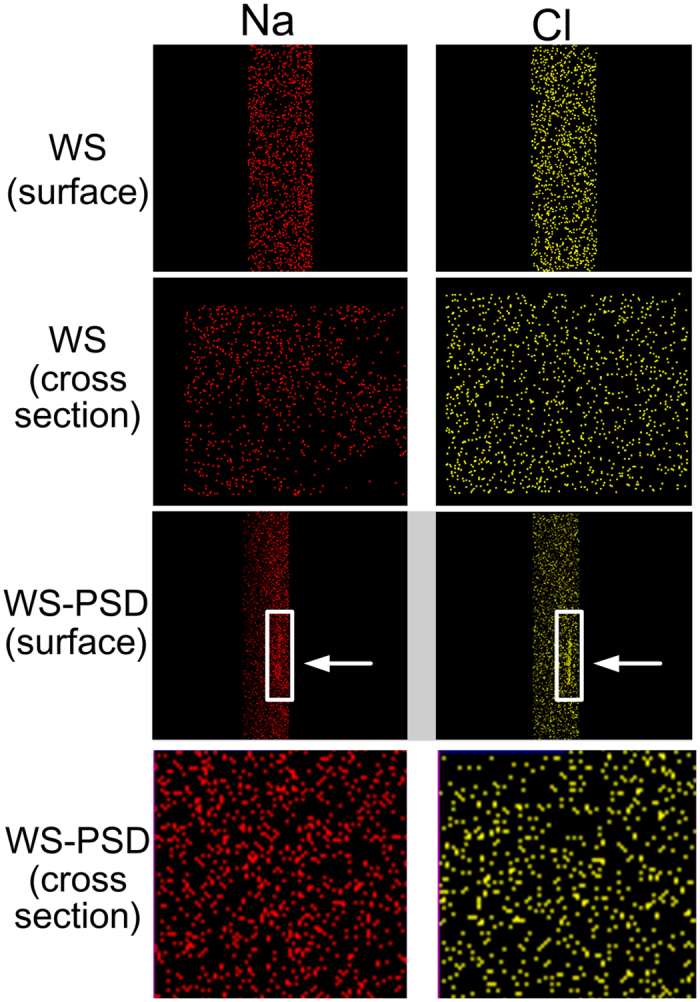
EDS element maps. Na and Cl on surface and cross-sections of recombinant spider silks. White arrows indicate NaCl aggregation on the surface of WS-PSD.

Energy-dispersive X-ray spectroscopy (EDS) element maps for Na and Cl indicate NaCl to aggregate on the surface of WS-PSD, while it distributes uniformly on the surface of WS (Fig. 7). These element maps of Na and Cl of the fibre cross-section reveal that NaCl disperses inside the fibres.

**Figure 8:**
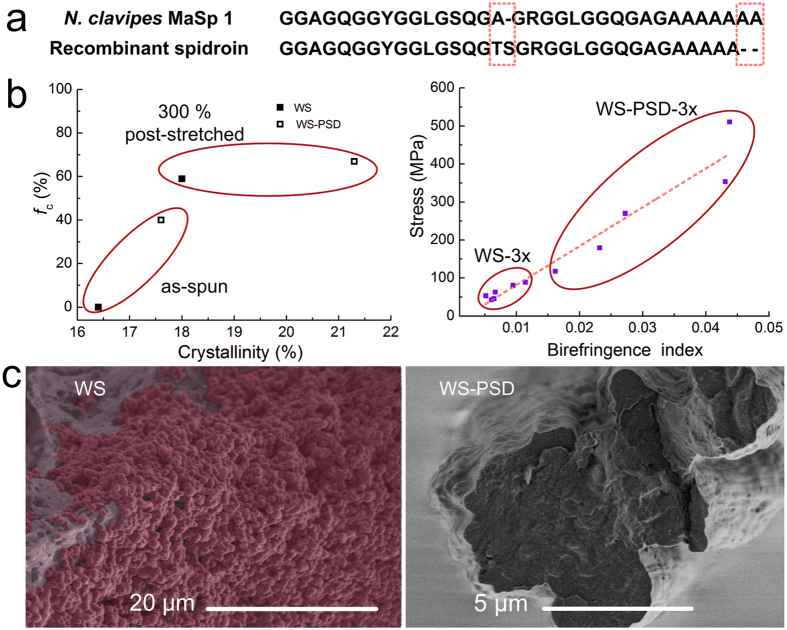
Hieratical structures of the recombinant spider silks prepared via wet-spinning and WS-PSD. **(a)** Repeat amino acid sequences of *N. clavipes* MaSp 1 (major ampullate spidroin 1)^6^ and recombinant protein^12^. Dashes indicate gaps inserted into alignments of ensemble repeat units for natural and recombinant spidorins. **(b)** Post-stretching effect on crystalline orientation, crystallinity (left), and birefringence index (right). **(c)** Cross section SEM images of as-spun WS (left) and WS-PSD (right) fibre.

However, Fig. 8b shows that the breaking strength of fibre correlates positively with birefringence, indicating substantial differences in molecular structures between those five fibres obtained from the same individual long fibre.

## Discussion

In addition to the important primary structure (Fig. 8a), the process of spinning is considered to be significant for the formation of other vital hierarchical structures, such as secondary structures, crystalline structures, and fibril structures. First, shearing and elongational forces supplied by microfluidics lead the orientation of microfibrils along the flow direction. Second, the crystalline and molecular structures greatly improved during the WS-PSD process. From Fig. 8b (left), we know that WS-PSD has a relatively high crystallinity and crystalline orientation, while WS is almost amorphous. Therefore, the dry-spinning step in air promotes the formation of an oriented β-sheet secondary structure. Fibrils are molecular networks with β-crystallites as nodes of the networks and molecular chains linking these nodes^35^. Post-stretching promotes the formation of a β-sheet structure. Furthermore, crystallinity and crystalline orientation of WS-PSD-3x and WS-3x both increased remarkably (Fig. 8b, left), while crystal size decreased following post-stretching. These changes in microstructure result in the formation of a rigid nanofibril. The orientation of molecular chains evidently increased for WS-PSD-3x. However, this effect is very limited for WS-3x (Fig. 8b, right).

Third, the spinning process considerably affected morphology and fibril construction structure of the fibres. As seen in Fig. 2a,b, as-spun WS fibres show apparent cracks and granules on the fibre surface as well as many irregular voids and pores in a cross section. The fibre formation process in ethanol coagulation mainly causes this, which is very different from the natural spinning process. *In vivo*, the epithelial tissues of the spider silk gland and spinning duct act as semi-permeable membranes, allowing water and sodium ions to leave the lumen, and potassium ions to enter the lumen^7^. Chaotropic ions have been proposed against the molecular assembly during fibre formation^16^,^36^. However, sodium and chloride ions cannot be removed during the *in vitro* wet-spinning process. A saturated NaCl solution promptly transformed into solid NaCl with the exchange of water in the dope and ethanol in the coagulation. A higher magnification of the cross section of WS illustrates globules with an average diameter of 1 μm (Fig. 8c, left). Considering the increased concentration in the spinning dope (NaCl:protein = 1:1 (w/w)), we assume the globules to be NaCl crystals. Consequently, precipitated or aggregated NaCl crystals in WS fibres or on the surface of WS fibrils could inhibit the assembly of fibrils, resulting in incompact structures with porous defects in WS. As shown in Fig. 8c (right), however, the compact WS-PSD fibre shows a smaller diameter than WS and no globules were observed in the cross section. Subsequent to coagulation in ethanol, the fibre is composed of recombinant MaSp1, NaCl and ethanol. Since major MA silk (an alloy of Spidroins 1 and 2) and high content NaCl have high water sensitivity^37^, this fibre is also very sensitive to water. During the continuous 5 s post-drawing process, water molecules in air may penetrate and plasticize the filament protein. Thus, it is possible for protein molecules to reorient and reassemble due to the improved mobility. Moreover, WS fibril bundles may also slip in air. Consequently, the WS slippery fibril bundles structure was changed into a WS-PSD bulk network structure, which is compact and smooth with aligned fibril bundles.

Intermolecular interaction has been proposed as essential for fibril material properties and the inter-backbone hydrogen-bonding network provides a major contribution to material rigidity^38^. We suggest the compact WS-PSD-3x to have strong interactions between fibril bundles, and furthermore, that adjacent molecular chains move together under the stretching force. However, interactions between fibril bundles of incompact WS-3x are weak; thus, fibril is easy to slip under the stretching force. We suggest that the compact fibril structure is vital for the formation of hierarchical structures.

In this study, we investigated the microfluidic spinning of water-soluble recombinant spider dragline silk protein (47 kDa). For WS-PSD, post-spin drawing in air promotes formation of compact fibres with ordered β-sheet structure. Subsequent to post-stretching, the fibre was comprised of a hierarchical structure and the breaking strength and strain of post-stretched fibre has been shown to reach up to 505 MPa and 44%, respectively. This spinning process is economic and non-toxic, compared to traditional wet-spinning of recombinant spider silk. It also breaks the molecular weight limitation to reap artificial fibre with high strength. However, the fluctuation of mechanical properties still remains problematic. Decreasing NaCl may directly lead to a stable mechanical performance. The addition of amino-terminal and carboxy-terminal may help spinning recombinant proteins with higher molecular weight and low salt concentration.

## Methods

### Preparation of recombinant dragline silk proteins and spinning dope

The recombinant spider dragline silk protein has 16 repeats of a monomer and a predicted MW of 47 kDa, was produced in metabolically engineered *Escherichia coli*^12^. The resulting recombinant protein is water-soluble, without amino-terminal or carboxy-terminal. To maintain stability and solubility of proteins at high concentration, the aqueous spidroin solution was dialyzed in 150 mM NaCl aqueous solution. Subsequently, the dilute aqueous solution was concentrated via forced air flow^31^. During the concentration process, some NaCl crystals precipitated from the solution with total concentration of the protein and NaCl of 42 wt%. Most of the water evaporated and the remaining NaCl was saturated in the dope. One drop of the dope on a glass slide was weighted and then dried for 2 h in an oven at 105 °C. The weight percent of the remaining solid compared to the weight of the drop before drying is the total concentration of protein and NaCl. At least four repeated measurements were performed. The solubility of sodium chloride in 100 mL water is 35.7 g (*m*_n_) and the protein weight (*m*_p_) in the spinning dope can be obtained according to the equation (*m*_n_ + *m*_p_)/(*m*_n_ + *m*_p_ + 100) = 42%. Thus the concentrations of NaCl and protein in the spinning dope are 20.7wt% and 21.3wt%, respectively. The weight percent of NaCl is estimated to be high and up to 50wt% in as-spun WS fibres.

### Spinning Apparatus and Fibre Formation

A microfluidic spinning apparatus was designed to create shear forces by mimicking the shearing gradients in the silk gland of silkworms^31^. The width of the microfluidic channel decreases from an initial width of 2065 μm to the terminal width of 265 μm (see Fig. 1a). The spinning dope was injected into the microfluidic channel and further extruded into the coagulation bath. The wet-spun fibres (WS) were reeled at a speed of 3 cm s^−1^ and kept in the coagulation bath. However, WS-PSD fibres were reeled in air at the same speed (3 cm s^−1^) after being reeled out of the coagulation bath. Ethanol was used as the coagulation bath for both spinning processes.

### Mechanical Testing

Mechanical properties of the fibres were tested, via an Instron 5565 material testing instrument (Instron Ltd., High Wycombe, UK) with a load cell of 2.5 N, at (24 ± 1) °C and (50 ± 5)% RH. The extension rate was set to 2 mm min^−1^, using a gauge length of 10 mm. At least five measurements were performed per sample. Diameter and birefringence of the fibres were tested with a BX-51 polarizing microscope (Olympus, Japan), equipped with a U-CTB Berek compensator. The fibre diameter was obtained from optical microphotographs using more than ten points distributed along the fibre axis.

### Fourier Transformed Infrared Spectroscopy

Fourier Transformed Infrared Spectroscopy was measured with a Nicolet iN10 MX (Thermo Fisher, USA) with a resolution of 4 cm^−1^ at 25 °C and 50% RH. The Amide I region has been used to examine the secondary structure changes of protein^39^. Secondary structure elements were assigned by the deconvolution results of the absorbance spectra ranging from 1580 cm^−1^ to 1720 cm^−1^. A baseline was subtracted from the spectra and a set of Gaussian peaks was fitted to the absorption curve. Data analysis was performed using the PeakFit routine of the Origin software (Ver. 8.5, OriginLab Corp.). The deconvoluted peaks for all assigned spectra are the same peak positions: random coil and helix structure at 1650 cm^−1^, β-turn structure at 1678 cm^−1^, and β-sheet structures at 1623 cm^−1^ and 1697 cm^−1^.

### Wide-Angle X-ray Scattering

X-ray fibre diffraction was performed at the BL15U beamline of the Shanghai Synchrotron Radiation Facility. The X-ray beam wavelength (λ) was 0.07746 nm and the beam size on the fibre was 3 × 2 μm. A single fibre was fixed to the sample holder, with fibre axis normal to the X-ray beam. The sample-to-detector distance was 164.6 mm and the exposure duration was 70 s. Background measurements were performed with the fibre removed from the beam. The 2D WAXD patterns were analyzed via FIT2D (V12.077) software. The radial 1D profile was integrated as a function of scattering angle (2*θ*) in a range between 5° and 25°. The baseline was subtracted from the spectra and a set of Gaussian peaks was fitted to the 1D spectra via the PeakFit routine of the Origin software package. The degree of crystallinity was obtained via the ratio of *I*_c_ to *I*_c_ + *I*_a,_ where *I*_c_ is the sum of the integrated intensities of the (200), (120), (211), (022), (013), and (023) crystalline peaks and *I*_a_ is the sum of the integrated intensity of the amorphous halo. The crystallite size was calculated according to Scherrer’s formula using the FWHM (full width at half-maximum of the peak) of the (200), (120), and (002) reflections^11^,^40^,^41^. Furthermore, azimuthal 1D profiles were integrated as a function of all azimuthal angles. Orientation of the crystallites about the thread axis can be obtained from the azimuthal profile at the radial position of equatorial (120) and (200) peaks. The orientation factor *f*_c_ of the crystalline and *f*_a_ of the amorphous were calculated according to Hermans orientation function^40^,^41^.

### SEM Observation

SEM images of surfaces and cross sections of fibres were characterized using a Hitachi SU-8010 equipped with an energy dispersive spectrometer (EDS). SEM images of surfaces and fracture sections were taken at 1 kV. EDS element maps were produced at 15 kV. WS and WS-PSD fibres were frozen and fractured in liquid nitrogen to obtain fracture cross-sections.

## Additional Information

**How to cite this article**: Peng, Q. *et al*. Recombinant spider silk from aqueous solutions via a bio-inspired microfluidic chip. *Sci. Rep*. **6**, 36473; doi: 10.1038/srep36473 (2016).

**Publisher’s note**: Springer Nature remains neutral with regard to jurisdictional claims in published maps and institutional affiliations.

## Supplementary Material

Supplementary Information

## Figures and Tables

**Table 1:** Crystalline structure and secondary structure content of recombinant spider silk fibres.

Sample	*f*_c_	*f*_a_	Crystallinity (%)	*L*_hkl_ (nm)			Secondary structure content (%)		
				(200)	(120)	(002)	β-sheet	Amorphous & helix	β-turn
WS	—	—	16.4	—	3.2	2.2	27	55	18
WS-1x	—	—	15.8	—	3.3	2.0	34	48	18
WS-1x-h	—	—	19	3.4	3.3	1.7	36	43	21
WS-3x	0.59	0.02	18.0	4.0	3.3	1.6	37	39	24
WS-PSD	0.40	0.02	17.6	5.2	3.5	2.7	33	39	28
WS-PSD-3x	0.67	0.03	21.3	4.9	2.9	1.6	43	35	22

**Table 2:** Comparison of mechanical properties among recombinant and natural spider silk fibres.

Protein origin	Molecular weight (kDa)	Solvent/coagulation bath*	Breaking Strain (%)	Breaking Stress (MPa)	Modulus (GPa)	Toughness (MJ/m^3^)	No. of samples
MaSp1	47 (WS-3x)	Water/ethanol	3.5 ± 1.2	62.3 ± 17.2	4.0 ± 2.8	1.6 ± 0.9	5
MaSp1	47 (WS-PSD-3x)	Water/ethanol	18.3 ± 12.8	286.2 ± 137.7	8.4 ± 4.3	37.7 ± 28.8	5
TuSp1 and MiSp1	378	HFIP/water	10	308 ± 57	9.3 ± 3	—	5^14^
MaSp1	284	HFIP/methanol	15 ± 5	508 ± 108	21 ± 4	—	10^12^
ADF3	268	GdmSCN/75% IPA	110 ± 25	370 ± 59	4 ± 1	189 ± 33	10^15^
MaSp1 and MaSp2	65	HFIP & formic acid/IPA	56 ± 7	221.7 ± 11.0	6 ± 0.5	102.5 ± 13.6	10^32^
MaSp2	86.5	HFIP/IPA	181.3 ± 103.5	39.0 ± 7.4	1.6 ± 0.4	59.3 ± 37.2	20^42^
Flag and MaSp 2	58	HFIP/IPA	18.7 ± 17.9	101.7 ± 32.8	3.3 ± 1.4	18.8 ± 20.7	11^19^
MaSp1 and MaSp2	250–320	water	24 ± 8	1183 ± 334	8 ± 2	167 ± 65	Natural dragline silk^15^

^*^HFIP: hexafluoroisopropanol; GdmSCN: guanidinium thiocyanate; IPA: isopropyl alcohol.
