# Supplementary material for: Recombinant spider silk from aqueous solutions via a bio-inspired microfluidic chip
Source: Sci Rep. 2016 Nov 7;6:36473. doi: 10.1038/srep36473 (PMC5098227; doi:10.1038/srep36473)
Supplement: Supplementary Information [file srep36473-s1.pdf]

Recombinant spider silk from aqueous solutions via a bio-inspired microfluidic chip

Qingfa Peng[#,1], Yaopeng Zhang[, #, 1], Li Lu[1], Huili Shao[1], Kankan Qin[2], Xuechao Hu[1], Xiaoxia Xia [*,2]*

[1] State Key Laboratory for Modification of Chemical Fibres and Polymer Materials, College of Materials Science and Engineering, Donghua University, Shanghai, 201620, China

[2] School of Life Sciences and Biotechnology, Shanghai Jiao Tong University, Shanghai 200240, China

Supplementary Results

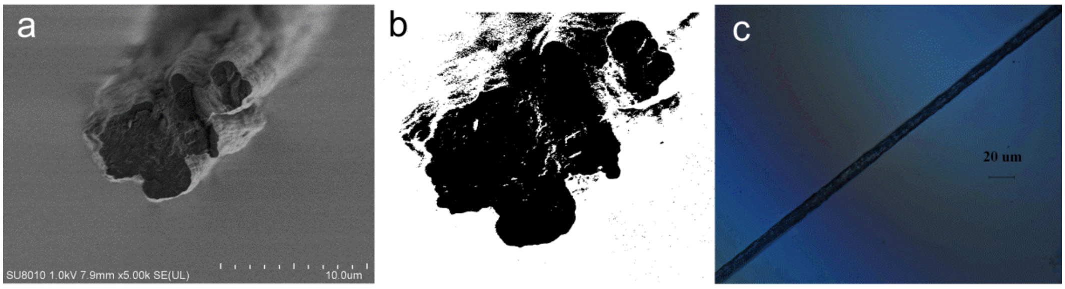

Supplementary Figure 1. Error of cross section area of recombinant spider silk fibres resulted from different measurements. (a) SEM image of cross section of WS-PSD. (b) Area of the irregular shape (dark region) was measured by software ImageJ (Area =55.2 μm2, Diameter =8.4 μm). (c) Diameter measured by polarizing microscope (area =59.4 μm2, diameter =8.7 μm).

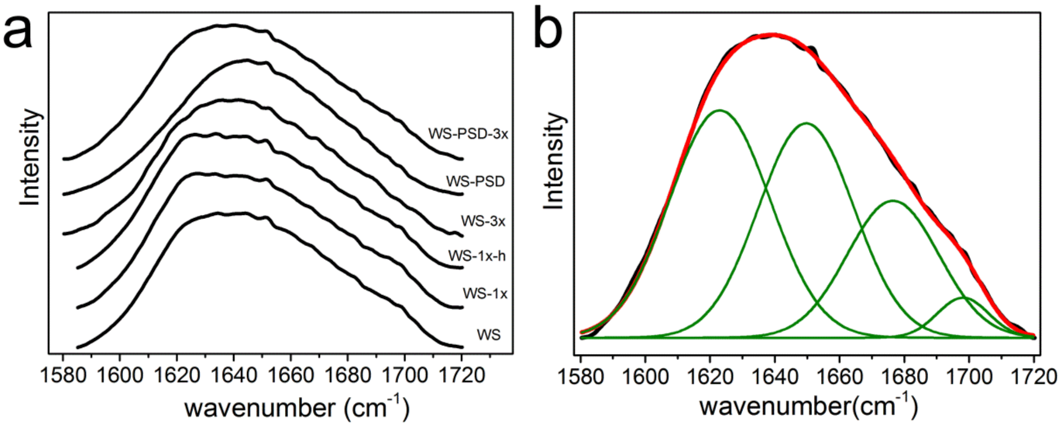

Supplementary Figure 2. Amide I region of infrared spectra of recombinant spider silk fibres. (a) After post-treatment and post-stretching, absorbance peaks of FTIR spectra clearly shift from 1645 cm^{-1} (WDS) to 1624 cm^{-1} (WS-PSD-3x), which indicates the formation of more β-sheet structures. (b) WS fiber is soluble in water and show mainly amorphous structure. Post-treated fibers of WS show more β-sheet structures. Typical deconvolution process of amide I region is illustrated in right and the detailed results are provided in Table 1.

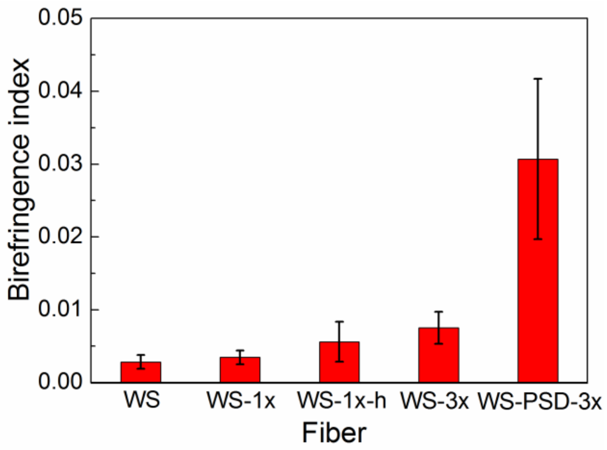

Supplementary Figure 3. Birefringence indexes of recombinant spider silk fibres. Data represent the mean and standard deviation of at least five filaments.

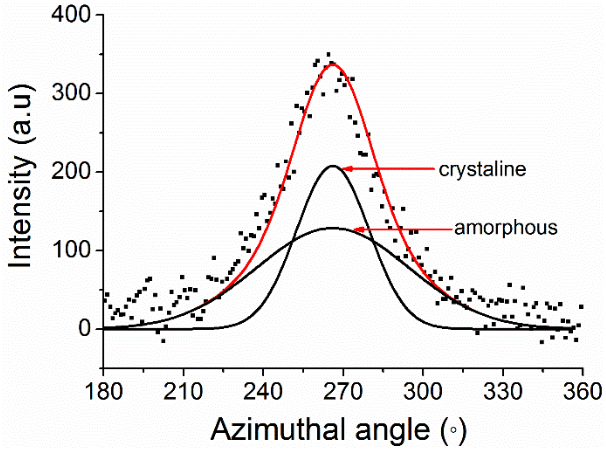

Supplementary Figure 4. 1D azimuthal intensity profile of the radially integrated (120) peak with Gaussian fits for WS-PSD-3X. The peak was fitted as sums of two Gaussians, corresponding to crystalline (narrow) and amorphous (broad) distributions. The orientation factor f_c of the crystalline and f_a of the amorphous were calculated according to Hermans orientation function.
